# The Care of the Teeth to the Fifteenth Year

**Published:** 1894-03

**Authors:** Safford G. Perry

**Affiliations:** New York City


### THE CARE OF THE TEETH TO THE FIFTEENTH YEAR.[1]

BY SAFFORD G. PERRY, D.D.S., NEW YORK CITY.

A LENGTHY monograph could be written on the care of the teeth up to the fifteenth year. I shall only attempt to consider briefly some of the most important points.

Commencing between the third and fourth years, I will go over the teeth somewhat in the order of their eruption.

In the successful management of little children there is a condition that precedes wisdom and antedates experience, and that condition exists in the heart rather than the head of the operator.

The rude instrument is tempered with gentleness and made a means of mercy in the hands of one who has a deep love for little children, and an operator who has little ones of his own, and whose heart in consequence is kept warm and sympathetic, will be more likely, all other conditions being equal, to be successful in saving the temporary teeth.

In caring for the temporary teeth, the heart should be full of love; in managing the permanent ones, the head should be full of wisdom. In other words, a cold, scientific method cannot be strictly applied to the care of the temporary teeth. The selection of filling-materials and the manner of using them may not be of much importance in teeth that are to last at the most eight or nine years, but in those that are to last a lifetime, the materials selected and the manner of using them may tax to the utmost the ripest powers of the best minds in our profession. It is doubtless true that many operators look with indifference upon the temporary teeth. This

---

[1] Read before the Odontological Society of Pennsylvania, January 13, 1894.

probably arises from the fact that these teeth, as the name implies, are temporary, and seem of less value, and while to work upon them is not exactly a waste of time, yet it is somewhat like writing in the sand,—a short time elapses and all trace is gone!

One can forgive a grown man for sometimes feeling impatient at having to spend his time excavating and filling little cavities in a baby's teeth, when he might be engaged in more responsible and important professional work; or when, with his experience of the world, and his knowledge of business principles, he might be at the head of some great business enterprise.

There are operators who take but little interest in children, and consider that until their permanent teeth need attention they are suitable patients for the assistant. They are annoyed by the timidity, the restlessness, the inquisitiveness of childhood, and persuade themselves that they are wise in reserving themselves for the permanent teeth. There are still other operators, and many patients who, considering the cost, and the pain to the little ones, mutually agree to reserve the labor and expense for the permanent set.

In a first-class city practice the question of expense is not, perhaps, a very important factor, but outside of the large cities it is important, and necessarily and justly must be considered in deciding upon the treatment of the temporary teeth.

In beginning with a family of children, it is well to have the little ones brought for an examination before there is much chance of work being required, so that they may go away with pleasant recollections of the operating-room. In fact, the whole training of a child with the temporary teeth should be preliminary to, and should lead up to, the proper care of the permanent teeth when they shall be erupted. A child well trained with the temporary teeth, which are not, as a rule, very sensitive, will be unconsciously well prepared to bear the harder work which will be needed in the permanent ones.

In operating for children, the first necessary condition is to get their confidence. When that is secured, the battle is more than half won. This will not be accomplished if the operator insists in all instances on doing thorough work. To excavate all cavities thoroughly, and to fill them accurately, may cause so much pain and may produce such an effect on the nervous system of the little patient, that the object sought—the salvation of the teeth—may be entirely defeated. The child, not yet old enough to be reasoned with and to exercise self-control, rebels, or, if of a patient nature and desirous of being submissive and obedient, gets into an hysterical state and makes a scene, and with the work unfinished is taken away by the parents, who declare that the teeth shall be lost rather than have their darling made so unhappy.

Instead of insisting, with the manner of a school-master, on thorough excavation, it is better to prepare the margins of the cavities with a light touch and a sharp instrument, cutting always slowly instead of rapidly and nervously, and, when necessary, leaving much of the softened and decayed tooth in the bottom of the cavity. If the margins are well prepared, and creosote or nitrate of silver is applied to this softened dentine, and the cavity is filled with oxyphosphate, decay does not progress to any great extent under the filling. At the end of six months or a year, when the filling is considerably worn down, it can be all taken out, and the cavity can then, in most cases, be painlessly excavated and filled in such a manner and with such materials as to last, perhaps, as long as the tooth will be needed.

In excavating cavities for little children, I go as far as I can without producing a state of great excitement, but rather than abandon the teeth I fill them in this imperfectly excavated condition, and generally with the results above described.

Sometimes, however, if a child from three to five years of age is brought to me with the conditions of the mouth so destructive and the teeth so badly decayed that they cannot be saved without an immense amount of work, and by keeping at work upon them until the permanent teeth come in, making a martyr of the little tot, I deliberately advise the parents to let them go unfilled. But I do this with the understanding that not only the teeth, but the roots also, shall not be extracted. I had rather have the child's mouth filled with decayed teeth and roots, accompanied, as they sometimes are, with disturbed gums and unpleasant breath, than have them taken out, and let the first permanent molars slip forward into their places.

Some of the most perplexing cases of irregularity I have ever seen have been produced in this way. I will not say that temporary teeth in front of the temporary molars may not sometimes be extracted, but I emphatically declare that the second temporary molars should not be taken out until the first permanent molars are in place and the second bicuspids ready to come in. I have no fear that even the diseased roots of the temporary teeth, if left in, will do harm to the second set. I have never seen such harm.

It may seem like shirking one's duty to abandon a set of badly decayed temporary teeth; but nature is not altogether unkind, and decay is so gradual and so unaccompanied by extreme sensitiveness in the temporary teeth, exposure of the pulps even being so often painless, or if painful so amenable to simple treatment, that I have little hesitation in advising the deliberate abandonment of teeth that are so badly decayed that the child must be made a martyr of if the attempt be made to save them.

A child with unfilled temporary teeth can be dismissed to the sea-shore or the mountains with very little danger of having pain which cannot be controlled by some simple remedy which any mother would know how to apply. But let a child with the temporary teeth all filled go beyond the reach of the dentist, and have a pulp die or a dead tooth ulcerate, and see how pitiable and helpless the case is. It makes the heart ache to think of the helplessness of a little sufferer under such conditions, and it should make one pause to ask if dentistry, after all, is an unalloyed blessing, practised as it is by so many who are without heart or judgment.

In making examinations, and in operating on the temporary teeth, one most important fact should be kept in mind,—namely, that, if possible, no cavity should be allowed to become so large as to expose a pulp.

This should be looked after in order to avoid the danger of future abscesses, as well as to insure the natural absorption of the roots on the approach of the permanent teeth. Of course it is well known that the roots of dead teeth are not absorbed, as is the case with the living ones.

If a pulp which has not been aching becomes exposed in excavating, I promptly cap it in the manner so long since described by Dr. Jack,—filling a little saucer-shaped cap of platinum with a paste made with the oxide of zinc and carbolic acid and oil of cloves, equal parts, placing this over the exposed point, and then filling the cavity with oxyphosphate. The metal cap prevents pressure, and the paste sterilizes the exposed point and allows healing by first intention. These little caps, as you know, are made by rotating a round-faced burnisher with pressure on a piece of thin platinum, which rests against a piece of soft wood. They are then cut out with a pair of curved scissors, and kept on hand of different sizes, ready for use.

If the pulps have been long exposed and aching, then I destroy them in the usual way; but after their removal I do not undertake to fill the roots to their extremities with any solid filling-material. It is quite likely that in this respect my practice is not like that of most operators. In the first place, I do not believe in filling roots unless it can be done so perfectly as to stop all germ action; and in the second place, I do not like to put in a filling that cannot be easily taken out. I take out the pulp and its canal extensions as well as I can, and then fill the canals, and sometimes even the pulp-chamber, with cotton saturated with iodoform. Occasionally I use oxychloride of zinc instead, but I do not mix it so as to make a hard filling, but only use a little of it entangled in the cotton so as to get its antiseptic effect.

In case of trouble, after the filling is removed from the cavity of decay, this cotton is easily pulled out of the roots, and relief usually ensues promptly. In impending trouble I have very many times passed a small bur under the gum on the buccal side into the pulp-chamber, but this is not very satisfactory if the cotton is closely packed, as vent is not secured.

In some unpromising cases I have put a piece of metal over the opening into the pulp-chamber, filled the cavity of decay, and then promptly drilled a small vent-hole from under the gum into the empty pulp-chamber.

Sometimes I have proceeded in this way, but have omitted drilling the hole, leaving that operation to be done only when needed. But in all cases where the hole is not drilled, I put into the pulp-chamber a few fluffy shreds of cotton carrying iodoform. The cotton is only used to convey the iodoform, and is so loose that if the tooth is ever drilled it does not prevent a prompt ventilation of the pulp-chamber.

In selecting a material for filling the ordinary cavities in the temporary teeth, there are several conditions that should be regarded. For instance, in cavities on the grinding-surfaces of the molars, for children who will not allow thorough excavation, I almost invariably use oxyphosphate. If the edges are fairly well prepared, this makes a filling which will usually arrest or delay the destructive action, even though considerable decayed dentine is left in the bottom of the cavity.

On the approximal surfaces I generally prefer gutta-percha for these partially prepared cavities, as it does not wash out near the gum. Sometimes, as with the permanent teeth, I use gutta-percha near the gum and oxyphosphate near the grinding-surface.

On the grinding-surfaces of the temporary teeth, if the cavities are large, the teeth of good quality, and the little patient will allow thorough excavation, I usually fill with amalgam, wiping away the surplus mercury from the surface of the filling with a little mass of well-annealed crystal gold.

If the cavity is small, so that not much surface is exposed to attrition, I fill with tin-foil. Under certain conditions I consider this the most beautiful material ever used for filling teeth. It is a constant wonder to me that the profession does not seem to appreciate its value. In searching for other materials its importance seems to have been overlooked.

Of course, I fill a great many of these large and small cavities with oxyphosphate, expecting to renew it when it has worn down.

On the approximal surfaces I am as careful about the restoration of contour as with the permanent teeth, for I consider it important that the little patient should be able to eat without the annoyance of the crowding of food against the gum. Occasionally I pack a single piece of gutta-percha into two adjoining approximal cavities, binding the two teeth together. The elasticity of the gutta-percha allows this, which would not be the case with the oxyphosphate or with amalgam. Either of the latter substances would break out from one cavity or the other, owing to the constant movement of the two teeth and the rigidity of the materials.

I quite often apply the rubber-dam to the temporary teeth, and when approximal cavities are to be filled, I find it of great value, not alone in keeping the cavities dry, but by allowing the use of different obtundents which can thus be kept off the gums. It also allows the neat use of nitrate of silver before filling. This I consider a preservative, as so well pointed out by Dr. Stebbins in the INTERNATIONAL DENTAL JOURNAL for October, 1891, though I use it somewhat differently from the manner described by him.

Long after I abandoned the Arthur system as applied to the permanent teeth, I continued its use with the temporary ones, but never except in cases where a firm point of contact could be left to hold the teeth in position and protect the gum. For many years I have abandoned all such cutting, except on the approximal surfaces of the temporary incisors, where I sometimes apply Dr. Arthur's system.

On all approximal surfaces of temporary molars I cut down, even for small cavities, from the grinding-surfaces, leaving as much as possible of the side walls standing, and then fill up flush, so that the perfect shape shall be preserved. I do not often cut in from the buccal or lingual sides, because the temporary teeth are, as a rule, very short. I take great care that no space is left into which food may crowd and be forced against the gum. Formerly, in cases presenting previous to the eruption of the first permanent molars, I often ground away the posterior surfaces of the temporary molars, leaving an abrupt shoulder at the gum, which was to hold the first permanent molar in place, but leaving its front surface free. This sometimes worked well, but not always by any means, for many times the first permanent molars would tip over against the cut surfaces and cause their decay, besides moving towards the front of the mouth and crowding out the bicuspids, which were yet to come.

After all these years of beating around the bush in the hope of finding some easier way to escape the dangers that beset the teeth, I have settled down to the belief that in developing a denture for a growing child the safest way is to follow the plan laid down by Mother Nature when she works at her best. In doing this, I keep two distinct objects in view,—namely, the preservation of the shapes of the teeth, so that comfortable use of them is assured, and the keeping of even the roots of the temporary teeth in position, so that the first permanent molars shall be held back in their proper places until the bicuspids are ready to come in. When all resistance or obstruction is removed, there is a marked tendency of the permanent molars to drift towards the front of the mouth, a fact which may be overlooked, particularly by young operators.

Dr. Dwinelle once told me that he had seen a first permanent molar which had drifted forward until it stood tightly against the cuspid. At the time the first permanent molars are coming in, if there is no obstruction in front, they drift forward with surprising rapidity.

In filling the approximal surfaces of the temporary molars, when the grinding-surface enamel has had to be cut away, leaving large openings into the cavities, I get a quick and excellent result by the use of hand matrices, which enable one to keep the shapes of the teeth, and which save the trouble of finishing off the fillings near the gum and on the entire approximal surfaces. I exhibited these matrices before the New York Odontological Society last April. They are kept in place by a handle which is held in the left hand, and they are of great value when operations have to be made quickly for restless patients. They are of equal value applied to the permanent teeth.

In preparing these cavities on the approximal surfaces for gutta-percha, it is well to avoid as far as possible cutting away much of the plate of enamel on the grinding-surface ends of the teeth, as this is of great value in protecting the fillings from rapidly wearing away. If gutta-percha is used in small pieces, and packed somewhat as gold is, it can be inserted through rather small openings, so that much loss of this enamel is not necessary. In such cases, of course, no matrix can be used.

Before leaving the consideration of the temporary teeth, lest I may be misunderstood, I want to repeat that I advocate the imperfect preparation of cavities only in cases where the teeth are very sensitive and the little patient is nervous and unmanageable, and when, if that humane plan were not followed, the task would be too great for both patient and operator. These imperfectly prepared cavities are greatly benefited by the nitrate-of-silver treatment.

We come now to the consideration of the permanent teeth. Naturally, according to the order of their development, we shall be concerned first with the lower first permanent molars.

With all families regularly under my care, I aim to have each child well in hand and in good training by the time these teeth appear. Without waiting for the teeth to get well through, I make a careful examination of all the fissures in the crowns. To do this, I often lift the flap of gum which commonly lies over the posterior portion of the grinding-surface with a peculiar instrument made for the purpose. If I find a soft place, even if it is not a pronounced cavity, I clear it out with a round-pointed instrument which is like a probe or examining instrument, and fill with oxyphosphate or tin-foil. I make very little attempt to cut out or prepare these little fissures, because at that time they are imperfectly calcified, and the tissue is so soft that quite a cavity could be easily made. A round-pointed instrument is all that is required for scraping out these fissures if they are to be filled with oxyphosphate.

A year later, when the tooth has grown out of the gum, and the filling nearly washed out, the border of the fissure will be so well calcified that a real filling may not be required. These teeth at this time do not antagonize with the upper molars, even if they are erupted, which they generally are not; so that the entire crown can be covered by the filling, and all its fissures protected, whether they show signs of softening or not.

For many years it has been my habit with soft teeth to wash out these fissures and put a pat of oxyphosphate in them, even if they are not softened at all. If they are dried out, the filling will usually stay, even if no cutting has to be done. This serves as a protection while the gums lie over the ends of the teeth, and while they are still unused in mastication. When the tooth gets well through, and is antagonized and used, the danger of softening of the fissure is greatly lessened. When the upper molars are through they are treated in the same way.

Later, after the lower molars are well developed, the little fissures on the buccal sides are to be carefully watched. If these are deep enough to catch the instrument, cavities must be made which will always have to be kept filled. There is no chance on this surface for the same kind of protection that can be given on the grinding-surface, pending the calcifying process. Here it must be a cavity for all time, or not at all. These little buccal cavities can often be filled in a few moments with tin-foil, in such a manner as to last many years. I almost never make any attempt at this time to fill them with gold, for the reason that I think I can fill them better with tin. If the child has not been brought soon enough, and cavities on the grinding-surfaces are of considerable size, I fill them almost invariably with tin or oxyphosphate. Sometimes, and for reasons which cannot be easily made plain here, I fill them with gutta-percha, but not very often with amalgam, and certainly not very often at this age with gold.

Although not quite in the order of time, it may be well here to dispose of the anterior approximal surfaces of these teeth. While the last temporary molars are in place these surfaces must be watched most carefully, and the slightest sign of decay must be regarded. I fill on these surfaces almost invariably with gutta-percha, as on all sheltered approximal surfaces. I believe this material will last longer than any other of the plastics. But when the baby molars are lost, and the anterior surfaces of the sixth-year molars are exposed, I almost invariably fill with gold, and in the most careful manner, and always strictly with regard to the natural shapes of the teeth, being careful to bulge the fillings out to a full contour, so that when the second bicuspids grow up they will rest against the fillings and as little as possible against the teeth. This operation is often difficult to perform. It is the first serious one the child has been asked to submit to, but there is no escape from it, for never again will the surfaces of these teeth be so favorably exposed and the opportunity given for the performance of so perfect an operation. If left until the second bicuspid is in place, then, of course, the teeth must be separated, and it will not be possible to reach the entire surface in so satisfactory a manner.

For this operation the rubber-dam is of course applied, and after the actual decay is removed, the whitened, partially decalcified margins of the cavity are bevelled so as to allow of extending the gold filling out on all sides beyond the real margin of the cavity. If the entire front surfaces of these teeth can be covered with gold, of course they will be safer when the bicuspids are in place against them.

The performance of this operation may mark the first real encounter with the young patient. If there has been gentleness and leniency before, there must be firmness and thoroughness here. But of course the patient is old enough by this time to bear such an operation, and to understand its significance and importance, and it is not often that one cannot, with the use of such obtundents as we now have, perform this operation in a perfectly permanent manner.

It seems to me that whatever may be said in favor of plastics before the fifteenth year does not apply with equal force to these approximal surfaces. In the first place, as before mentioned, they can never again be so well reached; and in the second place, they have been standing during several very dangerous years in contact with the baby molars, and if not actually decayed, their surfaces are likely to be more or less softened and in a condition favorable to decay soon after the bicuspids are in place. Gold is the only material that can be carried out to a thin edge over these bevelled margins with any certainty of its staying where it is placed. I instruct all parents to promptly bring their children when the posterior baby molars are lost. If they are careless about usual examinations, I beg them to be careful about this.

On the buccal surfaces of the first permanent upper molars, cavities sometimes occur which extend around partly on the posterior approximal surfaces. These cavities I usually fill with tin or gold before the appearance of the second permanent molar, and also for the reason that the posterior surface can never be so easily reached after the latter molar is in place. The posterior aspect of these buccal surfaces is very apt to be decayed before the second permanent molars are in place. The careful polishing of these surfaces, if promptly done, coupled with special directions for careful brushing, will often save them from decay.

"You must brush your teeth more," is advice that, given in the usual way, generally goes in one ear and out of the other, with the average patient of this age. But often a few earnest, impressive words, expressed with tact, will work wonders with some patients. The young mind is impressionable, and doubtless we do not always realize the effect a few earnest words may have. But they must not be expressed with the manner of a school-master. If the young patient is complimented with the assumption of possessing intelligence, and that intelligence is appealed to in a persuasive, convincing way, the seed may be sown in productive soil that will bring forth more than a hundred-fold. And this education of our young patients to an appreciation of the value of proper care of the teeth is as much our duty as to fill their teeth properly. I am sure most of us are too negligent on this point.

We must now go back several years to take up the superior permanent incisors. When these teeth are decayed on the approximal surfaces, one is brought face to face with a serious problem. The question is, To what extent shall gutta-percha, oxyphosphate, or tin-foil be used? I must say to you frankly that in most cases I use one or the other of them in preference to gold. Of course, I shall expect to be criticised by many for this, but I must be governed by the results of my own observation, and I have seen so many cases where gold fillings have so quickly failed, particularly when made soon after the teeth are erupted, that I have grown very shy about the use of gold at this time. At this age the teeth are so soft that even little cavities have to be enlarged to get the firm margins required for gold fillings, and when failure comes there must follow a still greater enlargement, so that by the time the child has arrived at twelve to fifteen years of age, by which time several renewals may have been required, large cavities exist.

If, instead, the teeth, when softened and slightly decayed, are opened with a separator, polished with a fine sand-paper strip, the little cavities cut out, *not too thoroughly*, and filled with a good quality of gutta-percha, or sometimes with tin-foil, and several renewals of these fillings made up to the fifteenth year, the chances are that the cavities will not be as large as if gold had been used. If as large, it is safe to say they will be no larger. There is still the advantage that not so much work has been done, and not so much expense incurred. Of course, if the teeth are of first-class quality, this does not hold true to the same extent as in those that are very soft, and in mouths that are destructive. The failure of a gold filling necessarily implies increase in the size of the cavity. The failure of a gutta-percha filling does not, by any means, imply such an increase. It fails from waste of substance of filling, and not from increase of cavity, and the same is true, to some extent, with a tin-foil or an oxyphosphate filling.

I have come to have very pronounced opinions on this question of selection of materials for young, soft teeth. Having had to manage a large practice for many years, I have often been forced from lack of time to adopt this plan of treatment for temporary purposes, but I have seen such good results from it when applied in this way that I have come to regard it, under certain conditions, as the very best practice for permanent purposes. I could show you cases where the bicuspids and molars are contoured with gold in the most elaborate manner, and yet on the approximal surfaces of the incisors only gutta-percha has been used for the last ten or fifteen years. Many renewals of the gutta-percha have, of course, been made in all this time, but there has not been an increase in the size of the cavities, and the teeth present a better appearance than they would if filled with gold. These fillings are in the mouths of most particular patients, who expect the very best treatment. The silk is used daily, the approximal surfaces are kept clean, and decay does not recur around these fillings. In making the renewals, only a few moments are required. A separator is applied, and space enough gained for passing a thin emery silk strip for finishing. After the filling is inserted, a piece of floss silk wet in chloroform is passed between the teeth, and the thing is done. Why should I fill these teeth with gold, when year after year shows no increase in the size of the cavities, and the patients greatly prefer to have them as they are? Of course, these are extreme cases, but they serve to show what can be done when great care is applied to this kind of treatment.

The next teeth in order are the second permanent molars. The fissures in these I treat in nearly the same manner as those of the first permanent molars.

The next after these are the bicuspids. The fissures in the crowns of these I also treat in nearly the same manner,—that is, I fill them with oxyphosphate or with tin-foil, mostly with the latter. In cutting out and preparing these fissures, as well as those in the molars, I am careful not to make large holes. I usually cut them out with extremely fine excavators, and aim to make the fillings as small as possible. If I use the engine for these, it is with only the minim burs, which are of course the smallest in the market. The engine is a savage instrument, and there is great danger, in its use, of cutting larger holes than are necessary. The teeth are priceless structures, and should be operated on with a feeling akin to reverence. This feeling is not much encouraged by the use of these modern dental saw-mills, which in steady hands may be safe enough, but which in unsteady or unwise ones may do an immense amount of mischief.

If the teeth are of good structure and the margins of the fissures hard, I fill them with gold once for all. With these teeth, as well as with the second permanent molars, we are dealing with a child older and more manageable than when the first permanent molars first required attention, and there do not exist quite the same reasons for the use of plastics.

We come now to the approximal surfaces of the bicuspids and molars. As with the corresponding surfaces of the incisors, we have to meet a question not easily disposed of. To a certain extent, with cavities to the fifteenth year, I follow the same plan as the one I apply to the incisors. If the teeth are very soft and the cavities very little, I almost invariably open them with the separator, polish the approximal surfaces with the sand-paper, and cut out the decay and fill with gutta-percha or tin-foil, being careful to in no way injure or change the contour of the teeth. In doing this I cut in from the buccal or lingual side, or down from the grinding-border, though I avoid, if I can, going through the enamel on the grinding-surface.

If the tooth is of fair quality, and the cavity so large that the enamel on the grinding-end is already crushed in, I treat it somewhat as I do the anterior surfaces of the first permanent molars,—namely, fill promptly with gold in the best possible manner. Such cavities, when filled, would leave but little of the tooth standing in contact with its neighbor, and there would not be much danger of a renewal of decay.

The reason for adopting this course lies mainly in the fact that the grinding-surface being undermined and destroyed, there is no protection to the gutta-percha, and an attempt to follow this line of treatment would necessitate such frequent renewals that what might be gained in one way would be lost in another. Let me say here that I use the oxyphosphate very little on the approximal surfaces, and for several years, I may say, not at all without putting a layer of gutta-percha to the extent of about a quarter or a third of the cavity, next to the gum.

Let this point be noted carefully: Oxyphosphate is the most dangerous and treacherous filling that can be put in on an approximal surface, unless it is intended to be, and unless it is sure to be, for the most temporary purpose. In advocating this plan of practice pending the solidification of the teeth, if it involved the use of oxyphosphate alone, on large approximal cavities, I should be preaching a doctrine fraught with the gravest dangers. Oxyphosphate, rightly used, is one of the best materials we have, but, with copper amalgam, it is responsible for the loss of an innumerable number of pulps, owing to undetected and treacherous chemical solution and waste along the cervical borders.

The safeguard against the danger of copper amalgam is never to use a particle of it under any circumstances. The last use I made of it before I excluded it from the office entirely, was in the small fissures of the grinding-surfaces of the molars. It does well in these places, but it wastes away somewhat like the oxyphosphate, though not so rapidly, and it discolors the teeth. I cannot imagine a fissure that cannot be as well filled with tin-foil, even if it has to be done under water. Copper amalgam has caused me so much vain regret, and so much humiliation, that I cannot think or speak well of it for any place in the mouth. In its present state of uneven and uncertain manufacture, I think it best to exclude it entirely. The safeguard against the danger of oxyphosphate lies in the use of gutta-percha along the cervical borders. This combination makes a very good filling for the approximal surfaces in young, soft teeth, or for frail, badly-decayed teeth of any age. In these approximal cavities, which have lost the enamel on the grinding-surface border, I have often used gutta-percha alone, protecting it from rapid wear by plunging into it a warmed platinum pin from an old rubber tooth, the head of which has been beaten out flat, and given a somewhat triangular shape corresponding to the outline of the grinding-surface aspect of the cavity. This stops the wear, and many times gives a good result.

A gutta-percha filling is not treacherous. It fails by wearing away on the surface, and that very deficiency brings the patient back with the fear that a filling is out. But the operator can possess his soul in peace, knowing that there is no danger to the tooth, because the wearing away of the filling brings the patient back in ample time. Had it been a filling of oxyphosphate, the patient would have been sailing on a happy summer sea, unconscious of the rocks beneath, and when brought back to the dentist by an ominous growl it would then be found that it was too late to avert all the ills that follow the exposure of the pulp.

Before I leave this interesting subject of the development of the teeth,—for that is what it is, Nature supplying the underlying force, and the dentist giving it, as the case may be, wise or unwise direction,—let me say, that in all the operations I have described or hinted at, both on the temporary and permanent teeth, I have had one fundamental idea in mind, although perhaps not before so clearly expressed, and that idea has been the *perfect preservation of their natural shapes*. From this I allow no deviation except in the case of temporary incisors, and in a few instances upon the posterior sides of the last temporary molars, but not until the first permanent molars are in place. Only with these few teeth do I admit that there is any advantage to be derived from the Arthur system.

Now, to sum up: This paper may seem to be a plea for plastics. So it is, to a certain extent, to the fifteenth year. But there is great opportunity here for misapprehension. Please note that I do not advocate this to the same extent when applied to teeth of good quality, in mouths which are not what may be called destructive; and please notice also that I admit that it is a dangerous practice if carelessly applied. It begets an easy habit of putting off until to-morrow what might be done to-day, and with careless patients, who do not return for frequent examinations, it may result disastrously. But it contains a principle of practice of great value, and one which applies with peculiar force to the very best class of patients that can be found anywhere,—that is, patients who can always be depended on to come at stated periods of the year for careful examinations. I have never yet seen a patient so rich or so exacting that I have not, under certain conditions of the teeth, promptly applied it as I would and do for my own children.

Instead of being a careless practice, it is a most careful one, and is susceptible of conferring a great benefit on the patient, and of being a great relief to the operator. It saves pain and expense, and renders the practice of dentistry bearable to both patient and operator.

Patients often remark what a difference between the gentle practice of to-day and that of the time when they were children. It was then a nightmare; it is now not a pleasure, but it is at least bearable. Some patients, before they comprehend its advantages, may think it a careless, lazy practice, and some dentists who do not look below the surface, but run on in the old groove, may consider it a makeshift; but these are enlightened days, and it has been my experience that most patients have a great deal of horse sense, and when taken into one's confidence, and told in a few frank, honest, earnest words the reasons for this practice, they readily acquiesce.

At about the fifteenth year the teeth have become so hard that the question of the use of gold must be seriously considered. After this time many of the arguments I have made vanish into thin air. Yet I must say that I carry along quite a number of people in moderate circumstances for whom I apply this practice, about as described, until far beyond the fifteenth year, and some for whom I continue it indefinitely. When second childhood approaches and the senile condition appears, it applies again with equal force,—I might almost say, with greater force in some cases. Some one has said that there is an eternal fitness in things throughout the universe. Since teeth must decay, how fitting and fortunate for humanity that we have such beautiful materials as gutta-percha, tin-foil, and oxyphosphate! If I do not couple amalgam with these, it is not because I do not use it, or because I have not respect for it, but rather, perhaps, because I cannot take quite the same pleasure in its use. It is not one of my pets,—it is one of my necessities. I do not include gold, because that is peculiar, and stands by itself, unrivalled still when used in its proper time and place.

The work of developing a set of teeth for a growing child is the most satisfactory of any professional work of which I have any knowledge. The physician, bowed with the fearful responsibility of life or death, gropes more or less in the dark, uncertain if his medicines really kill or cure. His problem is so complex that there must always remain a doubt, and though he has all the wisdom of all the ages, his patients must finally die. The surgeon, with better opportunities for exactness, and with the inspiration that springs from the more than marvellous advances that have been made in these recent years, cannot always make his patients live. But the dentist, full of reverence for the way in which Nature works, and recognizing the hinderances that prevent the fulfilment of her perfect purpose, can, by his deft fingers and steady brain and loving heart, give such aid and direction to her efforts, that his life-work may be a perpetual blessing to those who intrust themselves to his care.

